# Regulation of inflammatory pathways by cannabigerol in the collagen induced arthritis model in rats

**DOI:** 10.3389/fphar.2025.1705962

**Published:** 2025-10-28

**Authors:** Monika Šteigerová, Michaela Sklenárová, Mykhaylo Bazyuk, Luděk Záveský, Petr Jelínek, Mahak Arora, Daniel Stránský, Tomáš Kučera, Bruno Sepodes, Miroslav Šoóš, Martin Šíma, Ondřej Slanař

**Affiliations:** ^1^ Institute of Pharmacology, First Faculty of Medicine, Charles University and General University Hospital in Prague, Prague, Czechia; ^2^ Department of Chemical Engineering, Faculty of Chemical Engineering, University of Chemistry and Technology, Prague, Czechia; ^3^ Institute of Histology and Embryology, First Faculty of Medicine, Charles University, Prague, Czechia; ^4^ Faculdade de Farmácia, Universidade de Lisboa, Lisbon, Portugal

**Keywords:** cannabinoids, cannabigerol, rheumatoid arthritis, CIA model, inflammasome

## Abstract

**Objectives:**

This study aims to assess the anti-inflammatory properties of cannabigerol (CBG) in collagen-induced arthritis (CIA) model in rats, and to determine which inflammatory signaling pathways it affects.

**Study design:**

Rats were randomized into four groups: placebo (PCB)–p.o. Treated with 1 mL of 0.9% saline once daily, CBG–p.o. Treated with 30 mg of CBG/day, glucocorticoids (GC)–p.o. Treated with methylprednisolone 0.5 mg/kg/day, and negative control (CO)–p.o. Treated with 1 mL of 0.9% saline once daily. CIA was induced in the PCB, GC, and CBG groups. The effect of CBG was assessed by clinical scoring, paw width measurements, ELISA, and analysis of gene (qPCR) and protein (Western blot) expression of selected inflammatory markers in blood and synovial membrane.

**Results:**

Clinical scores showed significant improvement in the CBG vs. PCB on day 29 and in the GC vs. PCB on days 24, 27, and 29. MMP-3 levels in serum were significantly reduced in the GC vs. PCB. CBG demonstrated a selective anti-inflammatory and immunomodulatory profile, notably through the downregulation of key signaling molecules such as TLRs, systemic NF-κB p65, STAT-3, and inflammasome-related components including NLRP1A, NLRP3, AIM2, gasdermin D, and caspase-1. It also reduced IL-1β and TNF expression during the early phase of disease and increased expression of the anti-apoptotic gene BCL-2.

**Conclusion:**

Our findings indicate that CBG modulates distinct components of the inflammatory signaling pathways, and its effects translated into significant improvement in clinical scoring based on swelling, erythema, stiffness in rat CIA model.

## 1 Introduction

Rheumatoid arthritis (RA) is one of the most common chronic inflammatory diseases. It primarily affects the joints and leads to synovial hyperproliferation and bone destruction. Current therapies only provide symptomatic relief and slow down the progression of disease, but do not offer a cure. Despite extensive research, the exact cause remains unclear, but genetic, environmental, and immunological factors contribute significantly to the onset and progression of the disease (Smolen and Aletaha, 2015).

The disease is accompanied by an imbalance between pro-inflammatory and anti-inflammatory cytokines. Key inflammatory mediators such as Tumor Necrosis Factor alpha (TNF-α), Interleukin (IL)-6, and IL-17 promote synovial hyperplasia, angiogenesis, and osteoclast activation (McInnes and Schett, 2007).

Collagen-induced arthritis (CIA) is a widely used standard preclinical model for RA, sharing key pathological and immunological features. Both conditions are associated with specific MHC class II alleles and joint inflammation involving activated macrophages, fibroblasts, T cells, and granulocytes. This indicates that CIA and RA involve similar mechanisms of delayed-type hypersensitivity and immune complex–mediated inflammation (Holmdahl et al., 1989; Steigerová et al., 2023).

The endocannabinoid system (ECS) is involved in the regulation of vital physiological processes, including pain, appetite, fear, memory, and inflammation. Therefore, modulation of this system could represent a new therapeutic strategy, although the detailed interplay between ECS and other pathophysiological pathways in the etiology of RA are not well understood.

Studies in animal or human subjects have shown that within the ECS activation of the cannabinoid receptor CB2 mediates immunosuppressive effects by reducing TNF-α levels and modulating the Th1 helper response (Malfait et al., 2000). Limited evidence suggests that agonists of the CB2 receptor may mitigate joint inflammation, cartilage degradation, and bone destruction through mechanisms involving MAPK and Nuclear Factor kappa-light-chain-enhancer of activated B cells (NF-κB) pathways in the CIA (Turcotte et al., 2016; Kinsey et al., 2011; G et al., 2015; Nass et al., 2021).

Previous studies on potential anti-inflammatory effect of cannabinoids, focused mostly on cannabidiol (CBD) (Sklenarova et al., 2023), however, other non-psychoactive cannabinoid–e.g., cannabigerol (CBG) may be of interest as well. CBG has been observed to interact with a variety of target proteins, including α2-adrenergic receptors, the serotonin 5-HT1A receptor, peroxisome proliferator-activated receptor γ (PPARγ), cannabinoid receptor 2 (CB2), and transient receptor potential TRPA1 channel (Li et al., 2024; Brierley et al., 2016).

Based on this literature, the anti-inflammatory properties of CBG may be assumed, however, its impact on inflammatory pathways are not fully described. Thus, our exploratory *in vivo* study aimed to assess the anti-inflammatory properties of CBG in CIA model rats and to describe its effects on signaling pathways involved in the pathophysiology of CIA/RA.

## 2 Methods

### 2.1 Animals

Thirty-two female Wistar rats (250–300 g) were obtained from Velaz (Prague, Czech Republic) and housed under standard conditions (22 °C ± 2 °C temperature, 50% ± 10% relative humidity, and a 12-h light–dark cycle). Animals had free access to water and food during the whole experiment. All experimental procedures adhered to the guidelines for the use of animals set forth by the First Faculty of Medicine at Charles University. The study protocol received approval from the Ministry of Education, Youth, and Sports of the Czech Republic (MSMT-26838/2021-4).

### 2.2 Reagents

Bovine type II collagen and incomplete Freund’s adjuvant (Chondrex) were used for induction of CIA. Methylprednisolone sodium succinate (SOLU-MEDROL 40 mg, Pfizer), CBG nanoemulsion and 0.9% saline (placebo) were used for treatment. The tested CBG formulation was prepared as published previously (Ryšánek et al., 2025). Shortly, CBG (Pharmabinoid) was dissolved in the oil phase containing 50 wt% of Kolliphor EL (BASF, Germany), 30 wt% of Diethylene glycol monoethyl ether (Gattefossé, France), and 20 wt% of Propylene glycol monocaprylate (Gattefossé, France). Next, four parts of water with respect to one part of oil (by weight) were added dropwise to the oil mixture while mildly stirring prior to application. The final concentration of CBG was 15 mg/mL. The resultant nanoemulsion had a size of 32 nm, as measured using dynamic light scattering (LS Instruments, Switzerland).

### 2.3 Design of experiment

Collagen emulsion for CIA induction was prepared according to the Chondrex protocol by emulsification of incomplete Freund’s adjuvant with collagen type II bovine collagen dissolved in 0.05 M acetic acid (Chondrex and Inc, 2015). An initial 0.2 mL emulsion dose was administered subcutaneously at the base of the tails of adult female rats on day 0. A 0.1 mL booster injection was given 7 days later (day 7). Rats in the control group were injected with equivalent volumes of saline. After 30 days of therapy, the animals were euthanized with T-61 (Intervet International, B.V. the Netherlands).

On day 0, rats were randomized into four groups: placebo (PCB), cannabigerol (CBG), methylprednisolone (GC), and a control group (CO). The CBG group (n = 8) was treated orally by gavage tube with 30 mg of CBG in 2 mL of nanoemulsion once daily, GC group (n = 8) received an oral dose of 1 mL solution containing 0.15 mg methylprednisolone once daily, while PCB group (n = 8) and CO group (n = 8) were treated orally with 2 mL of saline solution once daily.

The severity of arthritis was assessed using a clinical score every other day. A score of 0 indicated an unaffected limb; a score of 1 indicated swelling or redness in one joint. A score of 2 was assigned if swelling or redness was present in more than one joint; a score of 3 indicated entire paw swelling and a score of 4 represented maximum swelling, erythema, stiffness affecting the ankle and distal digits. The clinical score of a rat was defined as the sum of the scores for each limb, with a maximum total score of 16 points. The ankle edema was assessed by measuring the width of both hind limb ankles with digital calipers on the day of randomization (Day 0) and at the end of the experiment (Day 29). On day 16, blood was collected for real-time polymerase chain reaction (qPCR) analyses.

After 30 days of therapy, all animals were euthanized, and their blood, ankles and synovial membranes were collected. Expression levels of cytokines and related molecules in blood and synovial membranes were evaluated by real-time polymerase chain reaction (qPCR), proteins were analyzed by Western blot (WB).

### 2.4 Sample preparation

Blood samples were mixed with an equal volume of Monarch DNA/RNA Protection Reagent (2X) and stored in aliquots at −80 °C according to Monarch Total RNA MiniprepKit (New England Biolabs) storage instructions. Synovial membranes samples were stored in RNAlater^®^ Stabilization Solution also at −80 °C. Blood samples for ELISA assay were centrifuged for 10 min (2,000 g, 4 °C), and serum was extracted. Serum samples were stored at −80 °C until analysis.

### 2.5 ELISA assay

Blood samples were taken on Day 29 to evaluate matrix metalloproteinase (MMP)-3 levels in serum. Blood was sampled from the heart immediately before euthanasia. MMP-3 levels were determined using Rat MMP3 ELISA kit (ab270216; Abcam, United Kingdom) according to manufacturer’s instructions.

### 2.6 RNA isolation, reverse transcription and qPCR

Total RNA from the whole blood samples was isolated by Monarch Total RNA MiniprepKit (New England Biolabs, United States) including DNAse treatment. Frozen synovial samples were homogenized, and total mRNA was extracted using mirVana miRNA Isolation Kit (Invitrogen, United States), DNase treatment was carried out with DNase I (1 U/μL) (Thermo Scientific, United States). Complementary DNA (cDNA) was synthesized using LunaScript RT SuperMixKit (New England Biolabs, United States). qPCR was performed on the QuantStudio 3 Real-Time PCR System (Applied Biosystems, United States) with SYBR Green Supermix reagent (Bio-Rad, United States) in a final reaction volume of 10 μL. Primers were designed using Primer blast (Ye et al., 2012). Housekeeping genes were selected based on relevant literature and validated genes included in the TaqMan Array Rat Endogenous Control Panel. The relative change in gene expression was analyzed by the 2^−ΔΔCT^ method, with RPL13A, B2M, PPIB and HPRT1 used as the reference genes for normalization. Primer sequences are listed in Supplementary Table S1. Due to the limited volume of blood available, the relative gene expression analysis on day 16 blood samples were performed only for a selected subset of genes.

### 2.7 Western blot

The synovial samples were homogenized and lysed in RIPA lysis buffer. The Pierce BCA Protein Assay Kit (Thermo Fisher Scientific, United States) was used to determine the amount of proteins, and the PageRuler Prestained Protein Ladder (#26616, Thermo Fisher Scientific, United States) was subjected to Mini-PROTEAN®TGX Stain-free. The proteins were separated using 4%-20% precast gels (#456-1093, Bio-Rad, United States) and then transferred electrophoretically onto a methanol-activated Wet Immobilon E (0.45 µm) nitrocellulose membrane. The membranes were blocked by incubating with Tris-buffered saline containing 5% bovine serum albumin (BSA) and 0.1% sodium azide for 30 min at room temperature. The membranes were then incubated with primary antibodies overnight at 4 °C. The primary antibodies utilized in this study are summarized in Supplementary Table S2. The following day, the membranes were washed in TBST buffer and incubated with goat anti-rabbit polyclonal IgG and horseradish peroxidase-conjugated antibody (1:10,000 dilutions, #ADI-SAB-300-J, Enzo Life Sciences, United States) or anti-mouse IgG (whole molecule)-peroxidase antibody (1: 10,000, #A9044, Sigma-Aldrich, United States) in 5% non-fat milk blocking solution at room temperature for 1 h. The protein bands on the membranes were then visualized via SuperSignal West Pico Chemiluminescent Substrate (Thermo Fisher Scientific, United States). The films were prepared in a darkroom, and the bands were detected using the FOMA LP-T and Fomafix (FOMA BOHEMIA Ltd., Czech Republic) treatment. The band intensities of target proteins were then quantified using ImageJ software (National Institutes of Health, Bethesda, United States). Finally, the band intensities were normalized to the respective house-keeping protein (β-2-microglobulin) and, subsequently, to the corresponding negative control group.

### 2.8 Statistical analysis

All data were expressed as the mean ± standard deviation (SD). The significance of the differences between groups was analyzed by one-way ANOVA with Tukey’s multiple comparisons test (comparison of all study groups) or by Student’s t-test (comparison of two selected groups). All statistical analyses were performed using Graphpad Prim 8.0; p-value ≤0.05 was considered statistically significant.

## 3 Results

### 3.1 Effects of the treatment on progression of CIA

**FIGURE 1:**
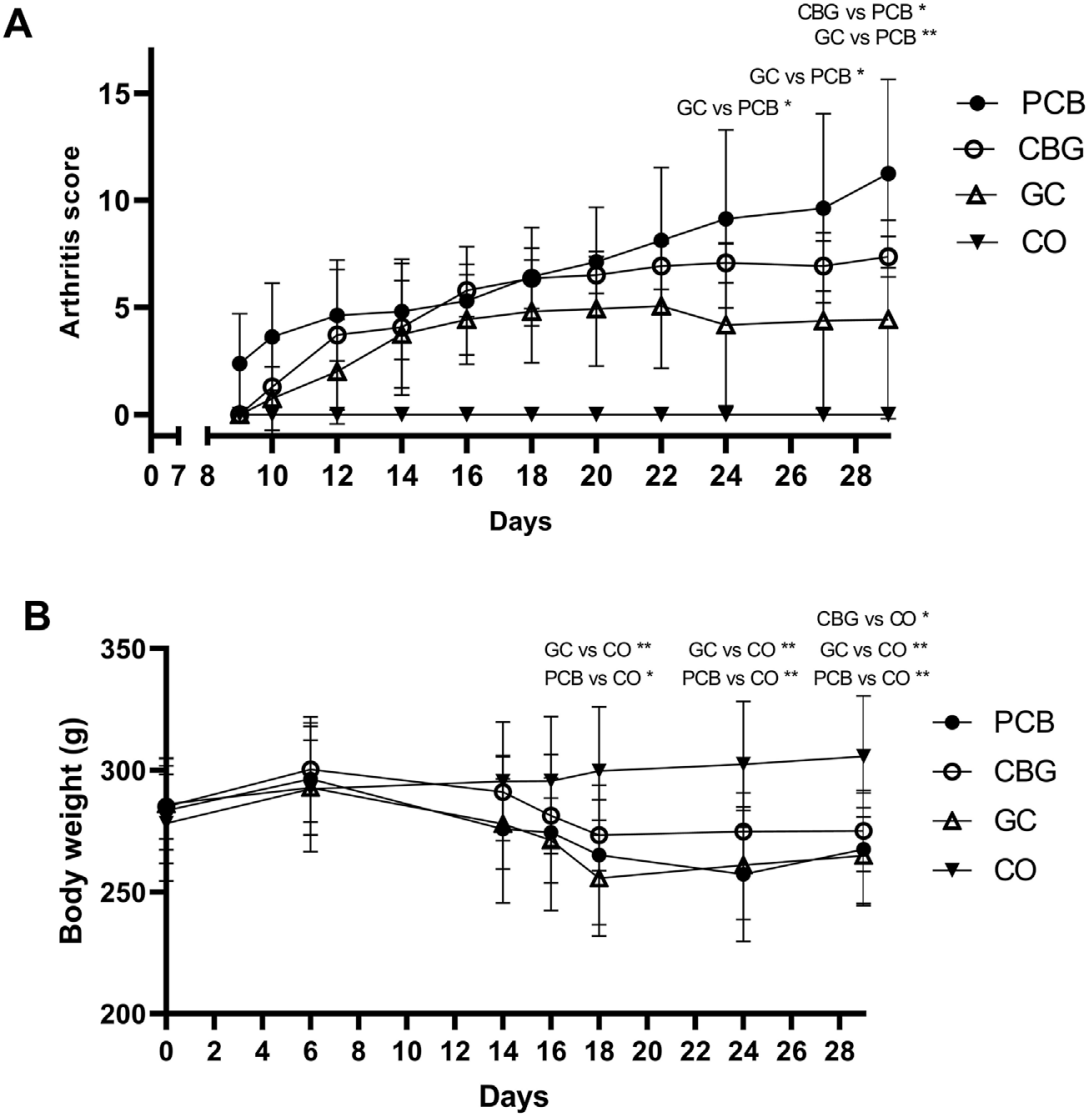
Clinical progression of collagen-induced arthritis expressed as arthritic score **(A)** and body weight development **(B)** in rats. Data are expressed as mean ± SD. CBG, cannabigerol group (n = 7); GC, glucocorticoid group (n = 8); CO, control group (n = 8); PCB, placebo group (n = 8). Statistical significance is indicated by * (p ≤ 0.05) and ** (p ≤ 0.01).

Clinical score and body weight development during the experiment is shown in Figures 1A,B, respectively. Ankle width is summarized in Table 1. PCB group showed significantly lower body weight (p = 0.0075), higher arthritis score (p < 0.0001) higher width of paws (p < 0.0001) and higher serum MMP-3 (p < 0.0001) compared to CO group, indicating that the CIA model was induced successfully. The arthritic score in the GC group was significantly lower than in the placebo group on days 24, 27, and 29 compared (p = 0.03, p = 0.03, and p = 0.009 respectively). The width of paws and serum MMP-3 levels in the GC group were significantly lower on day 29 compared to PCB (p = 0.002 and p = 0.0002, respectively). The arthritic score in the CBG group showed stabilization from day 18, reaching a significantly lower value than the PCB group on the last day (p = 0.04), although no significant reduction of width of paws compared to PCB group was noted. Significant weight loss was noted in all groups compared to the CO group; the CBG group experienced the least weight reduction.

**TABLE 1:** Mean ± SD values of ankle width and serum MMP-3 levels in rats.

	Ankle width (mm)				MMP-3 (ug/mL)
	Day 0		Day 29		Day 29
	Left hindlimb	Right hindlimb	Left hindlimb	Right hindlimb	
PCB group	6.5 ± 0.5	6.4 ± 0.4	9.4 ± 0.9	10.1 ± 0.7	708.0 ± 212.9
CBG group	6.4 ± 0.4	6.6 ± 0.2	10.0 ± 0.7	10.0 ± 0.4	701.3 ± 124.7
CO group	6.3 ± 0.3	6.5 ± 0.3	6.8 ± 0.4 ****	6.9 ± 0.4 ****	32.2 ± 7.5 ****
GC group	6.5 ± 0.2	6.3 ± 0.4	8.0 ± 1.1 ***	8.0 ± 1.1 ***	371.8 ± 107.3 ***

Mean ± SD values of ankle width, MMP-3 levels in rats. Results are expressed as the mean ± SD (CO n = 8, PCB n = 8, CBG n = 7, GC n = 8). Statistically significant difference vs. PCB group is indicated by *** (p ≤ 0.001), and **** (p ≤ 0.0001).

### 3.2 Effect on expression of toll-like receptors (TLRs) and inflammatory mediators

**FIGURE 2:**
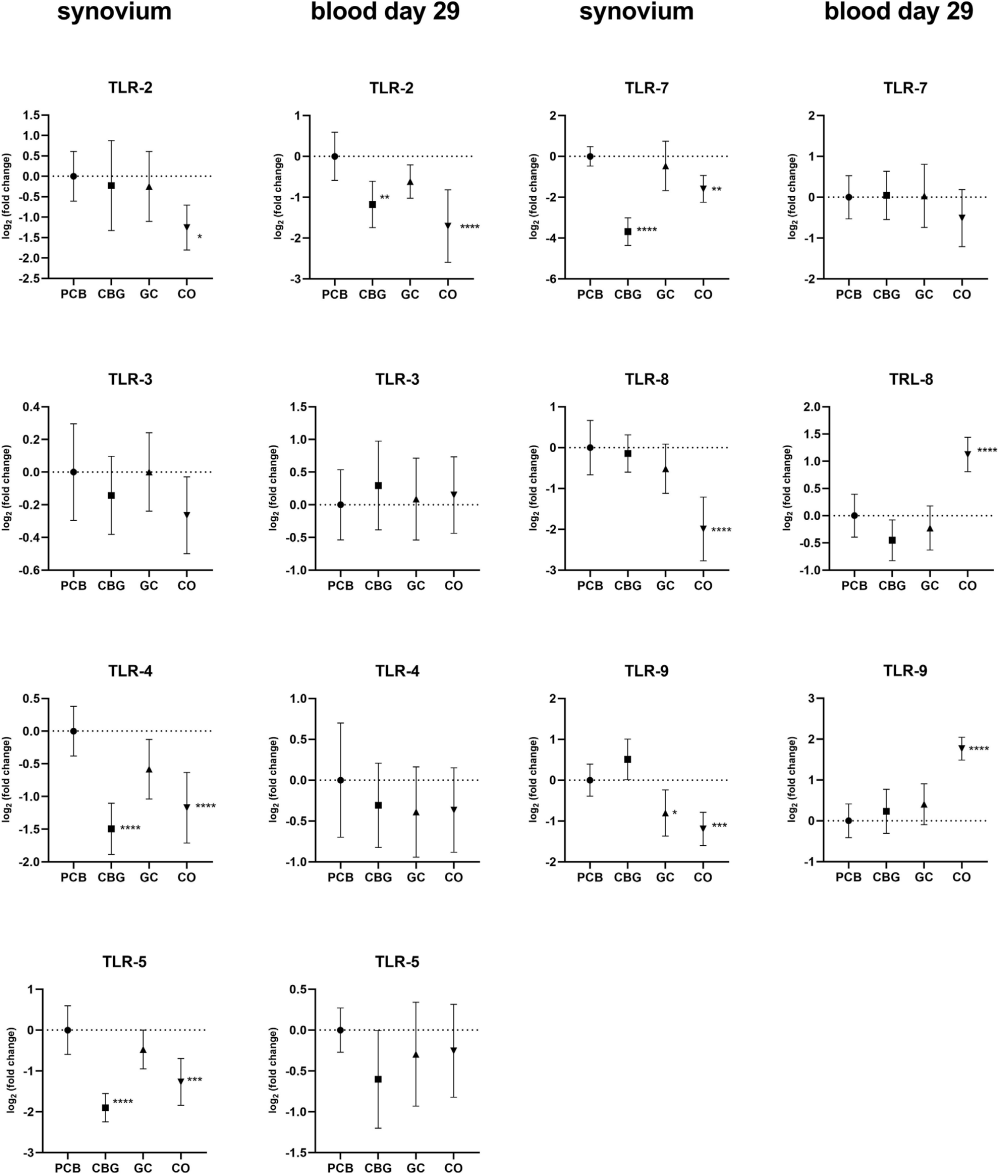
Effects of cannabigerol on relative gene expression of Toll-like receptors (TLRs) in synovial tissue and blood collected on day 29. Each graph shows the relative expression of one target gene in the cannabigerol 30 mg (CBG), glucocorticoid (GC), and control groups (CO) versus the placebo group (PCB). Results are expressed as log_2_ of fold change compared to PCB group, data represents the mean ± SD (CO n = 8, PCB n = 8, CBG n = 7, GC n = 8). Statistical significance is indicated by * (p ≤ 0.05), ** (p ≤ 0.01), *** (p ≤ 0.001), and **** (p ≤ 0.0001).

The greatest effect of CBG treatment on TLR expression was observed in the synovial tissue, with consistent trends generally noted in the blood on day 29 (Figure 2). TLR-2, TLR-4, TLR-5, TLR-7, TLR-8, and TRL-9 expression were markedly upregulated in the synovial tissue of the PCB group compared to the control group. CBG treatment significantly diminished the expression of TLR-4, TLR-5 and TRL-7 (p < 0.0001, p < 0.0001, and p < 0.0001, respectively), whereas GC treatment had no effect on these markers. CBG markedly decreased the expression of TLR-2 (p = 0.0072) on day 29 blood samples in comparison to both PCB and GC groups.

**FIGURE 3:**
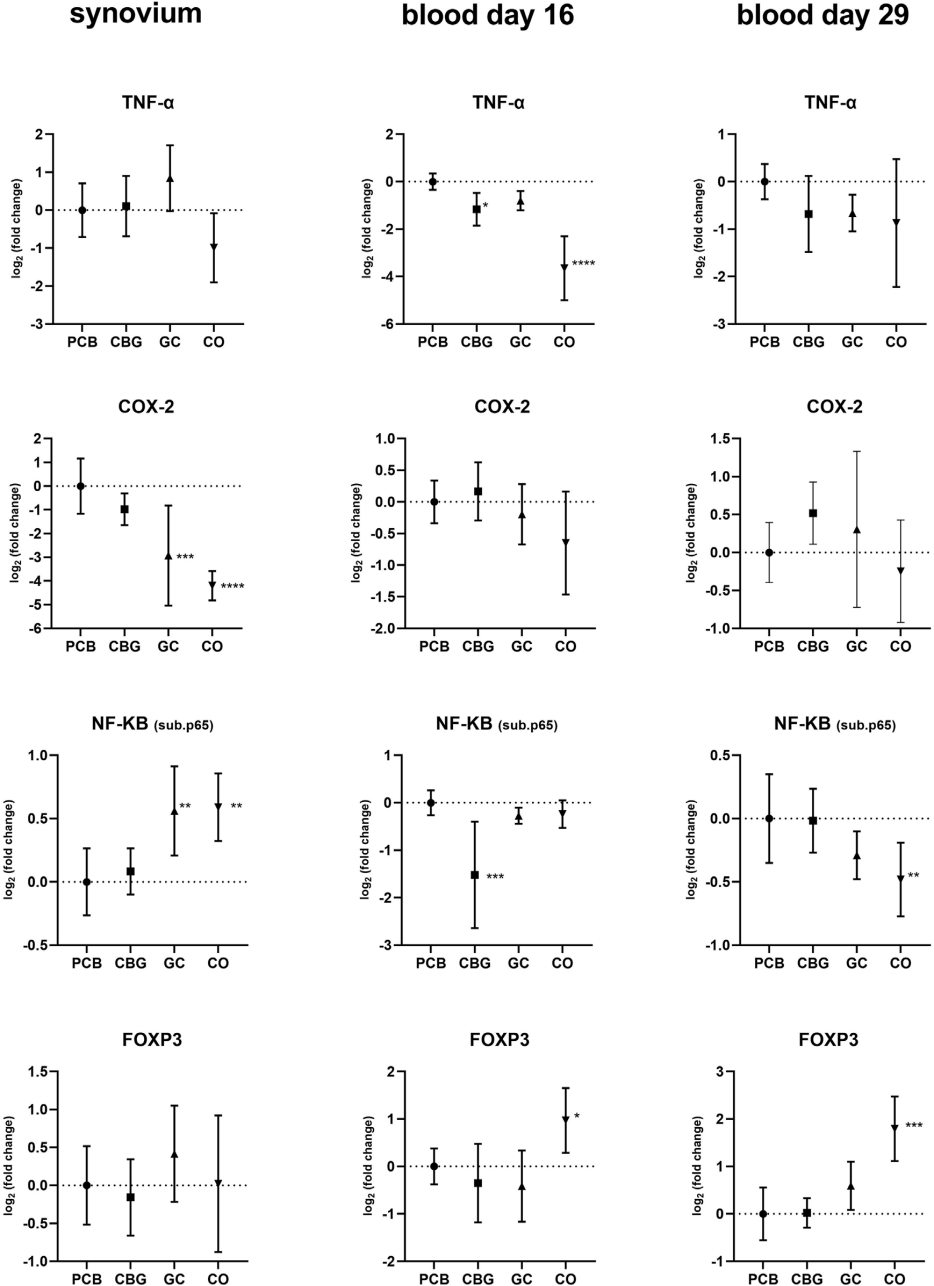
Effects of cannabigerol on relative gene expression of target genes in synovial tissue and blood collected on days 16 and 29. Each graph shows the relative expression of one target gene in the cannabigerol 30 mg (CBG), glucocorticoid (GC), and control groups (CO) versus the placebo group (PCB). Results are expressed as log_2_ of fold change compared to PCB group, data represents the mean ± SD (CO n = 8, PCB n = 8, CBG n = 7, GC n = 8). Statistical significance is indicated by * (p ≤ 0.05), ** (p ≤ 0.01), *** (p ≤ 0.001), and **** (p ≤ 0.0001).

Significant upregulation of NF-κB p65 subunit expression was observed in both CO and GC groups in synovium compared to PCB (Figure 3). Furthermore, CBG treatment did not significantly affect NF-κB expression relative to PCB. However, NF-κB p65 expression in blood on day 16 was decreased in the CBG group (p < 0.0001) compared to the PCB, while no effect was seen in the GC group. There were no significant changes in the NF-κB p65 expression in GC and CBG groups present on day 29.

**FIGURE 4:**
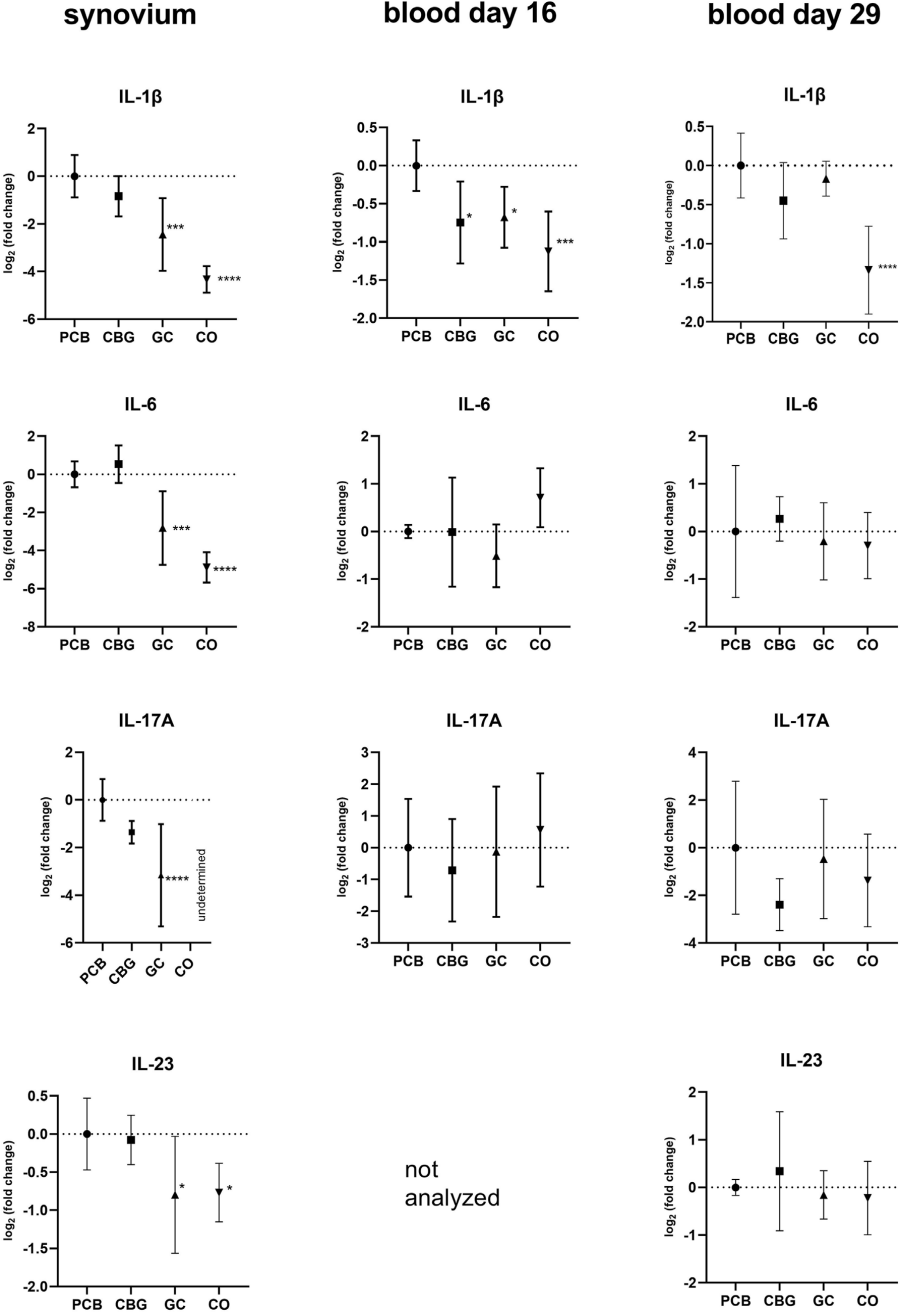
Effects of cannabigerol on relative gene expression of selected cytokines in synovial tissue and blood collected on days 16 and 29. Each graph shows the relative expression of one target gene in the cannabigerol 30 mg (CBG), glucocorticoid (GC), and control groups (CO) versus the placebo group (PCB). Results are expressed as log_2_ of fold change compared to PCB group, data represents the mean ± SD (CO n = 8, PCB n = 8, CBG n = 7, GC n = 8). Statistical significance is indicated by * (p ≤ 0.05), ** (p ≤ 0.01), *** (p ≤ 0.001), and **** (p ≤ 0.0001). Due to limited availability of blood material collected in the half of the experiment, relative gene expression analysis on day 16 blood samples were performed only for a selected subset of genes.

The pro-inflammatory cytokines TNFα, IL-17A, IL-23, and IL-1β exhibited elevated expression levels in all synovium samples and majority of blood samples of the PCB group compared to the CO group (Figure 4). GC treatment significantly decreased expression of IL-17A, IL-23, and IL-1β in the synovium compared to the PCB group (p < 0.0001, p = 0.0240, and p = 0.0003 respectively). Consistent with the NF-κB p65 expression pattern in the blood on day 16, CBG treatment reduced TNFα and IL-1β expression (p = 0.0438 and p = 0.0183, respectively) compared to PCB group, suggesting systemic anti-inflammatory effects at this time point.

Cyclooxygenase-2 (COX-2) expression was only affected by GC in synovium samples relative to the PCB group (p = 0.0007).

### 3.3 Effect on expression of inflammasome components

**FIGURE 5:**
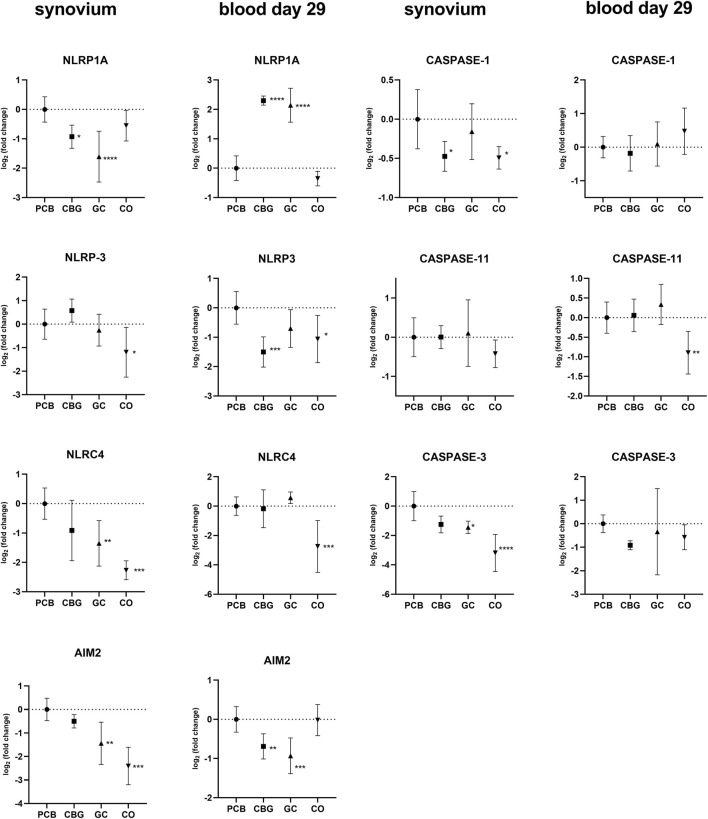
Effect of cannabigerol on relative gene expression of selected inflammasomes and caspases in synovial tissue and blood collected on day 29. Each graph shows the relative expression of one target gene in the cannabigerol 30 mg (CBG), glucocorticoid (GC), and control groups (CO) versus the placebo group (PCB). Results are expressed as log_2_ of fold change compared to PCB group, data represents the mean ± SD (CO n = 8, PCB n = 8, CBG n = 7, GC n = 8). Statistical significance is indicated by * (p ≤ 0.05), ** (p ≤ 0.01), *** (p ≤ 0.001), and **** (p ≤ 0.0001).

**FIGURE 6:**
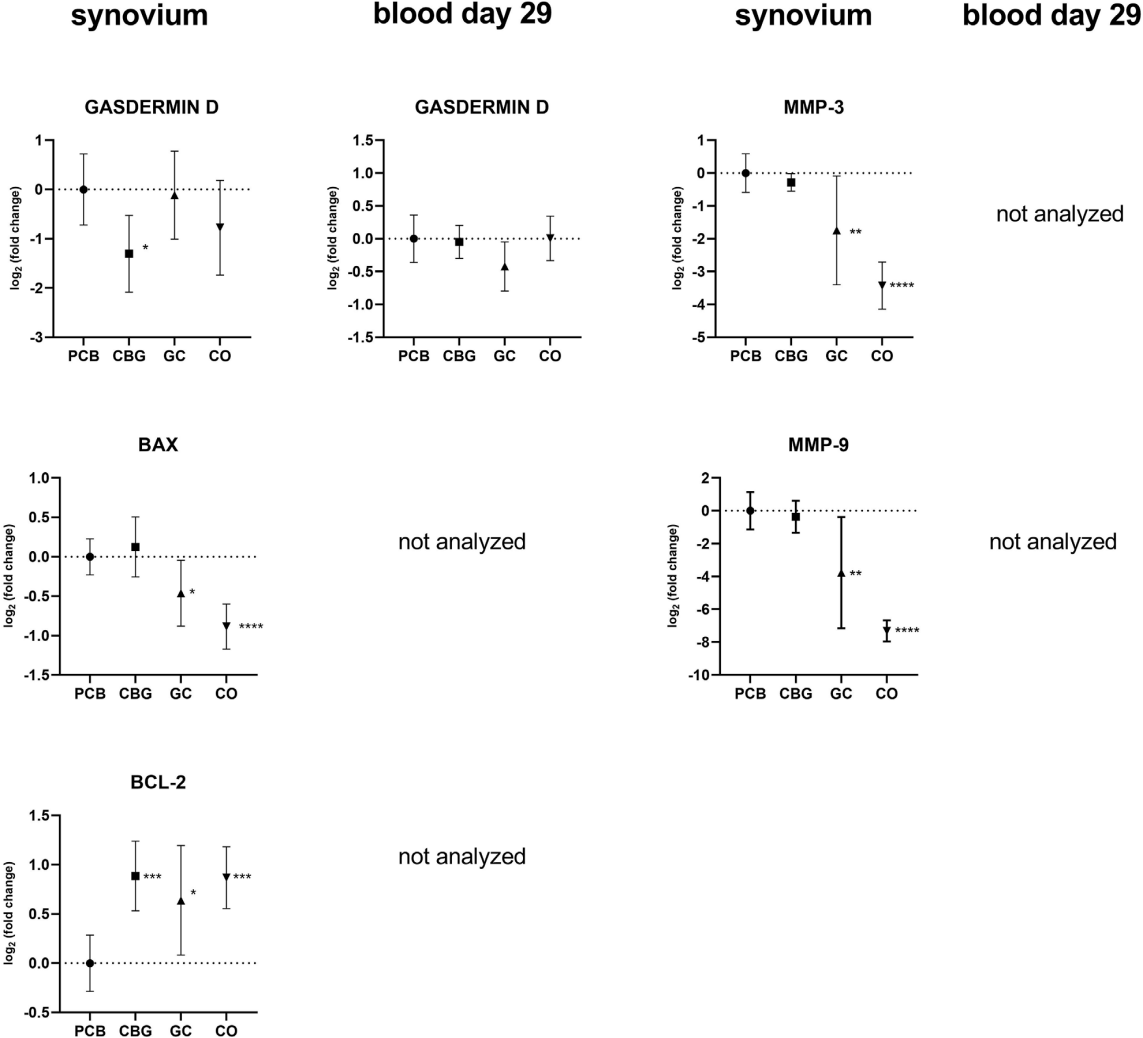
Effect of cannabigerol on relative gene expression of apoptosis factors and metalloproteinases in synovial tissue and blood collected on day 29. Each graph shows the relative expression of one target gene in the cannabigerol 30 mg (CBG), glucocorticoid (GC), and control groups (CO) versus the placebo group (PCB). Results are expressed as log_2_ of fold change compared to PCB group, data represents the mean ± SD (CO n = 8, PCB n = 8, CBG n = 7, GC n = 8). Statistical significance is indicated by * (p ≤ 0.05), ** (p ≤ 0.01), *** (p ≤ 0.001), and **** (p ≤ 0.0001).

The expression of inflammasome-related genes in the synovium, including NLRP3 (NOD-, LRR- and pyrin domain-containing protein 3), NLRP1A (NOD-, LRR- and pyrin domain-containing protein 1A), NLRC4 (NOD-like receptor family CARD domain-containing 4), AIM2 (Absent In Melanoma 2), caspase (Cysteine-aspartic acid protease)-1, caspase-11, and gasdermin D were elevated in the PCB group (Figures 5, 6). Treatment with CBG and GC led to a reduction in the expression of several of these markers. Specifically, CBG significantly diminished the expression of NLRP1A (p = 0.0247), caspase-1 (p = 0.0184) and gasdermin-D (p = 0.0288), suggesting an inhibitory effect on canonical inflammasome activation and pyroptosis pathways. In contrast, GC treatment significantly reduced expression of NLRP1A (p < 0.0001), NLRC4 (p = 0.0034), and AIM2 (p = 0.0011), which may reflect its broader suppressive effects on both canonical and non-canonical inflammasome activation pathways. Unlike CBG, GC did not affect the expression of either caspase-1 or gasdermin D. This suggests that CBG may specifically target the downstream effectors of pyroptotic cell death.

In blood samples collected on day 29, NLRP3 expression was significantly reduced only by CBG treatment (p = 0.0006) compared to the PCB group (Figure 5). The expression of AIM2 was significantly decreased in both CBG and GC (p = 0.0084, p = 0.0002, respectively) groups relative to the PCB. However, an unexpected increase in NLRP1A expression was observed in blood samples on day 29 in both CBG and GC (p < 0.0001, p < 0.0001 respectively).

### 3.4 Effect on expression of matrix metalloproteinases MMP-3 and MMP-9

As shown in Figure 6, the expression of MMP-3 and MMP-9 was not markedly affected by the CBG group. However, GC treatment significantly diminished the expression of both markers compared to the PCB group (p = 0.0069, p = 0.0026, respectively). These results are consistent with those from WB and ELISA analyses.

### 3.5 Effect on expression of janus kinase (JAK)/STAT signaling pathway

**FIGURE 7:**
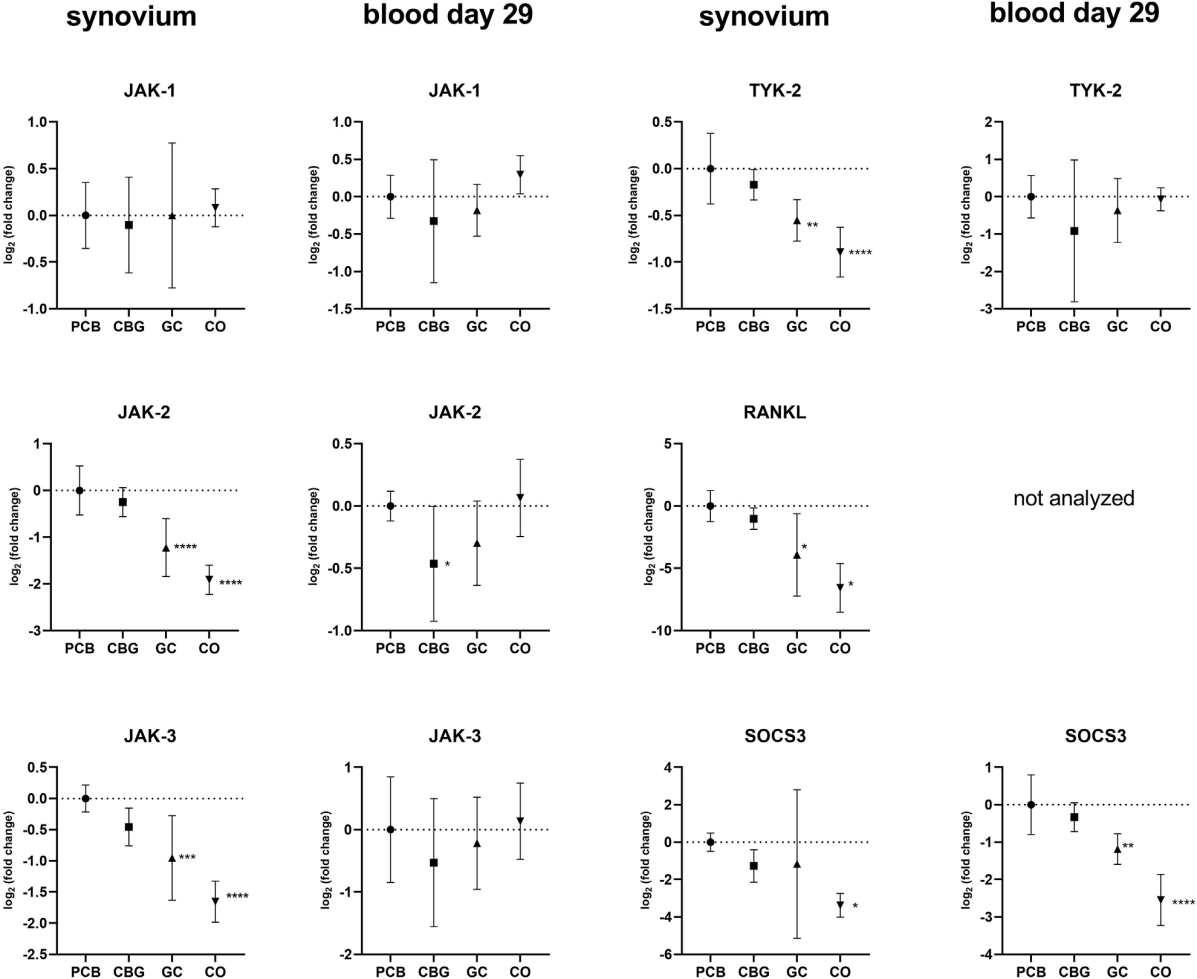
Effect of cannabigerol on relative gene expression of Janus and tyrosin kinases in synovial tissue and blood collected on day 29. Each graph shows the relative expression of one target gene in the cannabigerol 30 mg (CBG), glucocorticoid (GC), and control groups (CO) versus the placebo group (PCB). Results are expressed as log_2_ of fold change compared to PCB group, data represents the mean ± SD (CO n = 8, PCB n = 8, CBG n = 7, GC n = 8). Statistical significance is indicated by * (p ≤ 0.05), ** (p ≤ 0.01), *** (p ≤ 0.001), and **** (p ≤ 0.0001).

Treatment with GC significantly reduced JAK-2, JAK-3 and TYK-2 expression in the synovial tissue compared to the PCB group (p < 0.0001, p = 0.0006, p = 0.0020, respectively), but there was no effect of CBG (Figure 7). However, CBG significantly diminished expression of JAK-2 in blood samples on day 29 compared to the PCB group (p = 0.0474).

**FIGURE 8:**
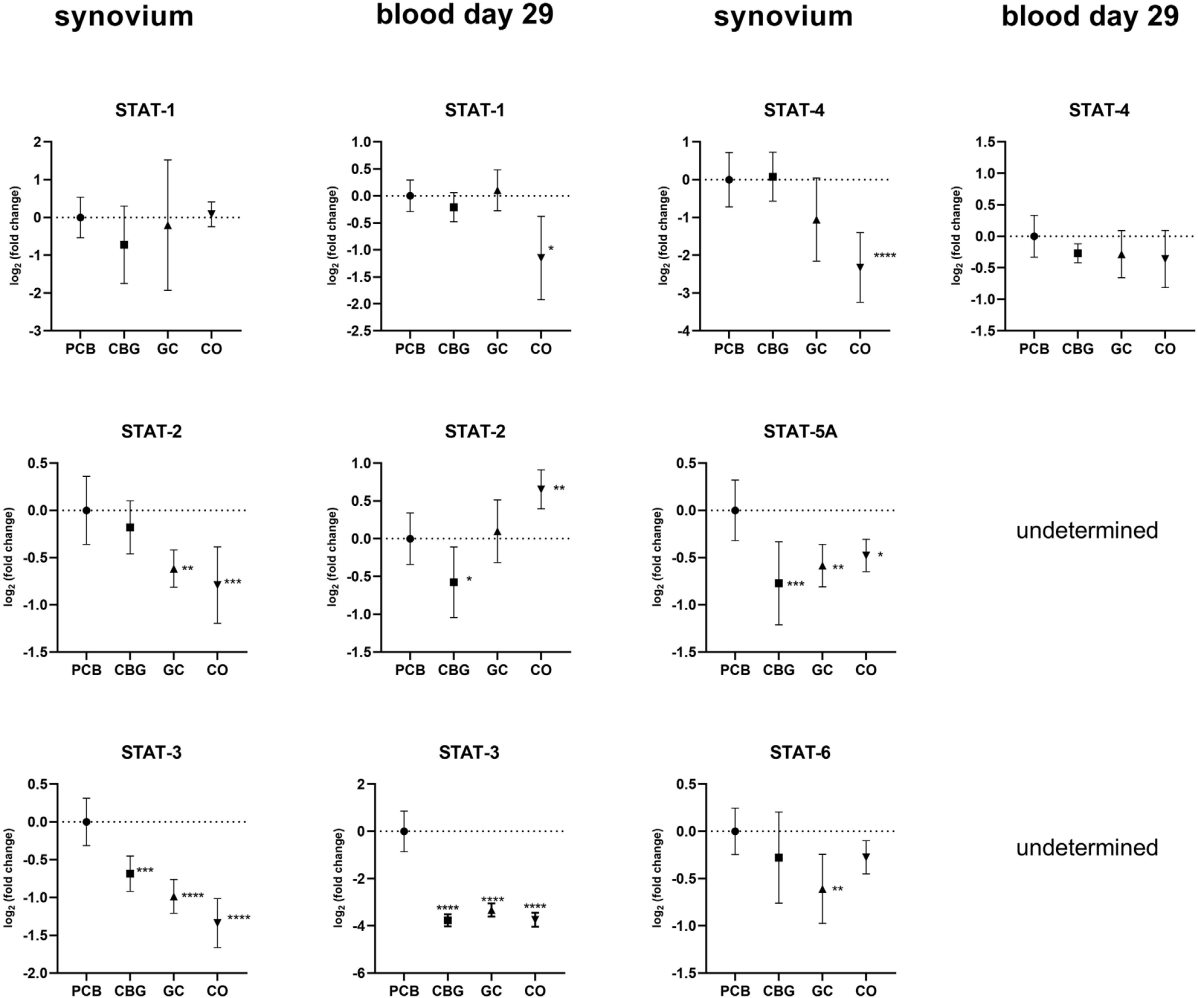
Effect of cannabigerol on relative gene expression of STATs in synovial tissue and blood collected on day 29. Each graph shows the relative expression of one target gene in the cannabigerol 30 mg (CBG), glucocorticoid (GC), and control groups (CO) versus the placebo group (PCB). Results are expressed as log_2_ of fold change compared to PCB group, data represents the mean ± SD (CO n = 8, PCB n = 8, CBG n = 7, GC n = 8). Statistical significance is indicated by * (p ≤ 0.05), ** (p ≤ 0.01), *** (p ≤ 0.001), and **** (p ≤ 0.0001).

The relative expression of STAT family genes was higher in the PCB group compared to the control group in synovial samples (Figure 8). Treatment with GC significantly decreased the expression of STAT2-, STAT-3, STAT5-A, and STAT-6 relative to the PCB group (p = 0.0037, p < 0.0001, p = 0.0031, p = 0.0054, respectively). In the CBG group, only the expressions of STAT-3 and STAT5-A were markedly downregulated in the synovium (p = 0.0003 and p = 0.0002, respectively). CBG also significantly reduced STAT-2 (p = 0.0291) and STAT-3 (p < 0.0001) expression in blood samples on day 29. A significant decrease in STAT-3 expression was also observed following GC administration in blood samples (p < 0.0001). The suppressor of Cytokine Signaling (SOCS)-3 expression was significantly downregulated in synovial tissue following GC treatment compared to PCB, with no significant changes observed in the CBG group.

### 3.6 CBG and its effect on apoptosis and cell death markers

Gasdermin D expression was reduced in synovial tissue only by CBG treatment relative to the PCB group (p = 0.0288) (Figure 6). Caspase-3 expression was significantly lower in the GC group (p = 0.0150), while CBG produced a similar but less pronounced reduction in synovium samples. Neither CBG nor GC administration produced significant effects on caspase-3 expression in blood compared to the PCB group.

The expressions of BAX (B-cell lymphoma 2-associated X protein) and BCL-2 (B-cell lymphoma 2) were not detected in blood samples, however, in synovial membranes, BAX expression levels were reduced in the GC group and BCL-2 expression was marginally increased in CBG, GC and CO groups compared to PCB (Figure 6).

### 3.7 Effect on proteins levels of pERK-1/2/ERK-1/2, p-STAT-3/STAT-3, TNF-α, IL-6, MMP-3 in synovial tissues

**FIGURE 9:**
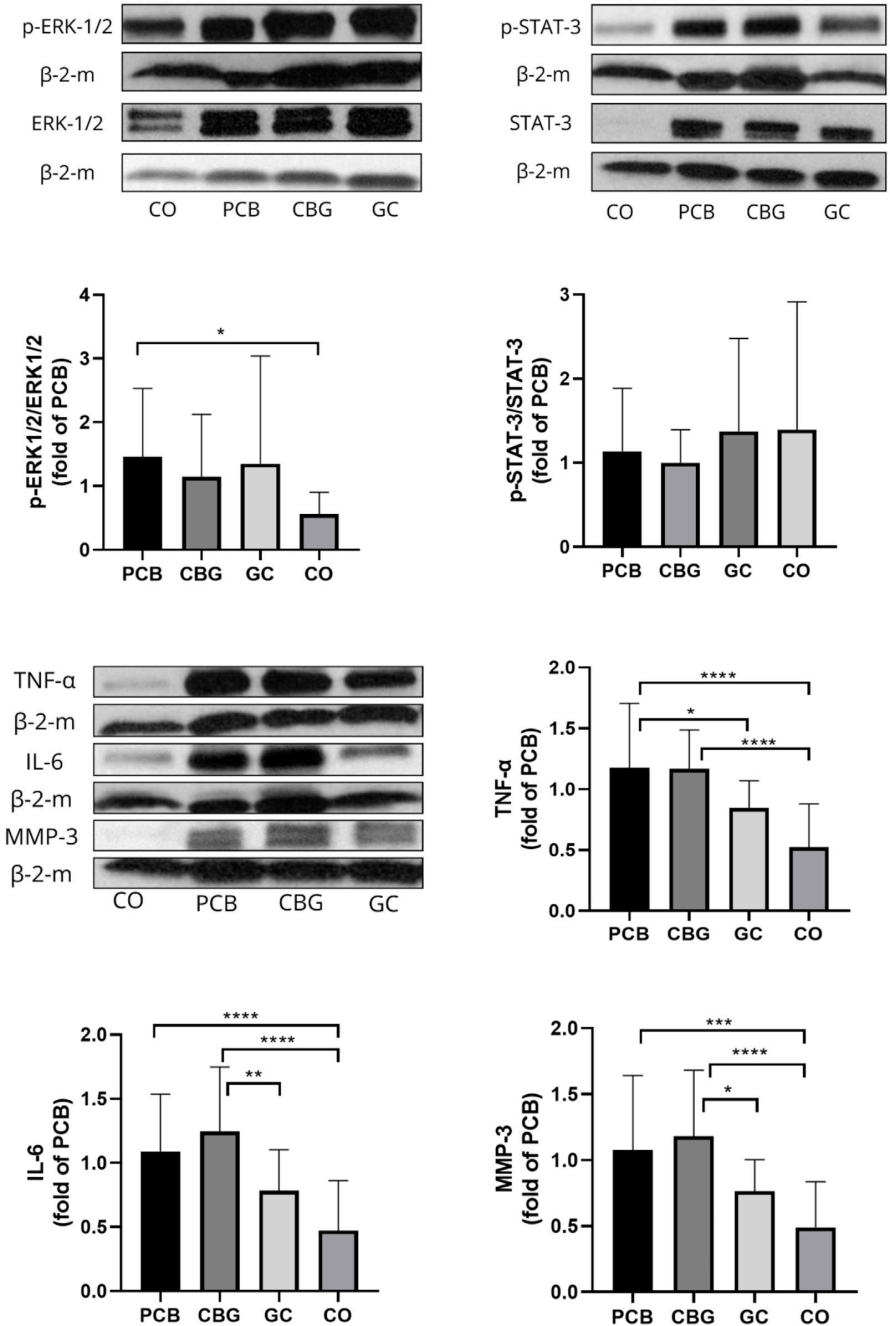
Proteins levels of pERK-1/2/ERK-1/2, p-STAT-3/STAT-3, TNF-α, IL-6, MMP-3 in synovial membrane in CIA rats in the cannabigerol 30 mg (CBG), glucocorticoid (GC), and control groups (CO) versus the placebo group (PCB). Representative imagines of Western blot protein bands in synovial membrane of CIA rats, Quantitative densitometry of proteins bands in synovial membrane of CIA rats β-2-m was used as a loading control. The data are expressed as the mean ± SD (CO n = 7, PCB n = 8, CBG n = 7, GC n = 6). Statistical significance is indicated by * (p ≤ 0.05), ** (p ≤ 0.01), *** (p ≤ 0.001), and **** (p ≤ 0.0001) compared to placebo group.

Phosphorylation of ERK was upregulated in the synovial membrane of CIA rats and in the treatment groups. Only a non-significant trend towards decreased protein levels of p-ERK1/2 and p-STAT-3 (Figure 9) was noted in the CBG group. Significantly decreased TNF- α (p = 0.0471) content compared to PCB group was seen in the GC group as well as a trend towards decreased MMP-3 and IL-6 protein expression (p = 0.1451 and p = 0.1055, respectively).

## 4 Discussion

In our study, CIA was reliably induced in rat model, as evidenced by clinical arthritic scores and paw thickness. The disease activity was also indirectly reflected by weight loss in the animals (Alabarse et al., 2018). The oral dose of CBG uses 30 mg per rat to ensure sufficient systemic exposure despite low bioavailability, staying within safe and effective ranges reported in literature (Sztolsztener et al., 2024; Weerts et al., 2024; Borrelli et al., 2013). The treatment effect of CBG was demonstrated in the clinical score at the end of the experiment, which was significantly lower than the placebo score. There was a trend towards delayed onset of clinical score increase, i.e., appearance of the disease symptoms in the CBG group. While the extent of CBG efficacy did not match that of GC according to the clinical score and paw volume, the CBG group experienced the least weight loss of all the groups in which CIA was induced. This may reflect the effect of CBG and its potential in stimulating appetite (Brierley et al., 2016).

The thickness of the paws in the CBG group is very similar to that in the PCB group, which suggests that edema and joint hyperplasia were not affected by CBG. Although paw thickness was similar, differences in arthritis scores arise because the scoring system evaluates all four limbs and individual joints, assigning points for localized swelling or redness that may not substantially alter overall paw thickness. These results are consistent with previous findings that CBG does not prevent acute inflammation (paw edema) or reduce pain hypersensitivity (hyperalgesia and allodynia) in carrageenan-induced hyperalgesia and mechanical pain sensitivity model in rats (Weerts et al., 2024).

Conversely, GC, which have well-demonstrated efficacy in RA, have previously been found to reduce bone destruction in the murine CIA model on mice based on X-ray analysis, which corresponds to our observations (Toonen et al., 2014).

In our study, CBG treatment significantly suppressed the expression of TLR-4, TLR-5, and TLR-7 in the synovial tissue, as well as TLR-2 in blood on day 29. In contrast, GC treatment had no effect on these receptors but was found to markedly decrease the expression of TLR-9 in synovium. TLRs signaling leads to activation of NF-kB, AP-1, MAPK pathway and interferon regulatory factors (IRFs) to induce pro-inflammatory cytokines as TNF, IL-1β, IL-6 and INF and it has been shown that altered TLR expression is closely associated with RA, as summarized recently (Botos and Davies, 2011; Kawasaki and Kawai, 2014; Cui Sun et al., 2024; Unterberger et al., 2021). Therefore, the observation of TLR expression suppression by CBG supports the mechanistic plausibility of the treatment effects of CBG as reflected in the clinical scoring.

Our findings support the existing hypotheses that cannabinoids act as key regulators of TLR signaling, consequently decreasing the expression of pro-inflammatory cytokines and the activity of transcription factor NF-κB (Cui Sun et al., 2024; McCoy, 2016). However, there has been no direct experimental evidence specifically evaluating the impact of CBG on TLR expression until now. In a study by Delgado-Arevalo et al., the authors reported that CBG significantly reduced TLR-2-mediated IL-1β release in human keratinocytes stimulated with *Cutibacterium acnes*, however, they did not directly measure TLR-2 expression at the gene or protein levels (Perez et al., 2022). Their proposed mechanism was inferred from published literature. In another study, the authors observed that CBG (alone or in combination with CBD) reduced TNFα expression (Mammana et al., 2019). The combination of CBG and CBD also prevented IkBα phosphorylation and nuclear translocation of NF-kB in a motoneuron cell line that had been exposed to medium from lipopolysaccharide (LPS)-stimulated macrophages. This effect could be associated with modulation of TLR-4, a key pathway activated by LPS. However, this conclusion was also based on prior literature and was not supported by direct measurement of TLR-4 expression or activity (Cui Sun et al., 2024; Mammana et al., 2019). Therefore, our findings provide novel and direct evidence of CBG-mediated modulation of TLR expression in an *in vivo* model of inflammation.

Regarding the effect of GC on TLRs, our findings are consistent with the established hypothesis that GCs primarily suppress inflammation by inhibiting the downstream TLR signaling pathways, rather than directly reducing TLR gene expression (Cain and Cidlowski, 2017).

The TLR-dependent activation of the NF-κB and MAPK pathways occurs via MyD88 (Myeloid Differentiation Primary Response 88) or TRIF (TIR-domain-containing adapter-inducing interferon-β) proteins (Cui Sun et al., 2024). Within the NF-κB pathway, we assessed the relative expression of the NF-κB p65 subunit, which forms the most abundant form of NF-κB heterodimer (p50 + p65) activated by the canonical pathway. Our findings suggest that CBG exerts a time-dependent systemic inhibitory effect on NF-κB p65 expression, which is evident in the early phase (blood, day 16) and may contribute to the observed reduction of expression of systemic cytokines such as IL-1β and TNFα (Figures 3, 4). These results are consistent with findings from a previous study investigating CBG in a mouse model of atopic dermatitis (Jeong et al., 2025). In that study, CBG led to downregulation of IκBα, NF-κB, and p-NF-κB protein levels, along with a reduction in IL-1β mRNA, supporting its anti-inflammatory potential through modulation of the NF-κB pathway.

Surprisingly, we observed a lack of significant modulation of NF-κB p65 expression in the synovium by CBG, which may be a result of limited tissue penetration, tissue-specific regulatory mechanism or represent time dependency of this effect. Interestingly, GC treatment led to increased NF-κB p65 expression in the synovium, despite its known role as a transcriptional inhibitor of NF-κB. This paradox could be due to compensatory feedback mechanisms, where the cell upregulates NF-κB components in response to inhibited activity.

Further, our focus was on inflammasomes, which are key components of innate immunity. The activation of these cytoplasmic multiprotein complexes has been shown to contribute to the pathogenesis of RA (Jiang et al., 2021). Although the anti-inflammatory properties of cannabinoids are the subject of an increasing amount of research, no published data currently exist that directly examine the effects of cannabinoids, particularly CBG, on inflammasome signaling in the context of RA. Existing literature includes data few other cannabinoids, but only in models of other inflammatory disease (Suryavanshi et al., 2021).

In our study, CBG significantly reduced the expression of NLRP1A, caspase-1, and gasdermin D in synovial tissue, suggesting a targeted inhibitory effect on canonical inflammasome activation and pyroptosis (Figure 5). In addition, CBG reduced the expression of NLRP3 and AIM2 in blood, which may be mediated by the modulation of TLRs, NF-KB and CB2 receptor. In a previous study, selective CB2 receptor agonist HU308 was found to inhibit the NLRP3 inflammasome in mouse peritoneal macrophages and an experimental colitis mouse model (Ke et al., 2016). Another study showed that CB2 receptor activation attenuated neuroinflammation through autophagy-mediated degradation of the NLRP3 inflammasome in an LPS-induced microglia inflammation model and a mouse model of spinal cord injury (Jiang et al., 2022a). The CB2 receptor antagonist AM630 produced opposite results.

The expression of inflammasome-dependent cytokine IL-1β was significantly decreased by both CBG and GC in blood samples collected in the early phase of the experiment (day 16), with a non-significant downward trend still observed at day 29 (Figure 4). In synovial tissue, IL-1β suppression was observed only with GC treatment. This observation could be caused by tissue-specific mechanisms or limited CBG distribution into the synovial tissue. The reduction of IL-1β and AIM2 expression by CBG in blood may be associated with its CB2 receptor agonism, potentially modulating the canonical way of NLRP3 inflammasome activation through the downregulation of TLR2, NF-κB and NLRP3 (Figures 2, 3, 5). The key role of AIM2 in RA have been described previously, and silencing of the mRNA AIM2 expression has been associated with diminished IL-1β production (Zhao et al., 2022; Chen et al., 2020). No data exists on the effect of CBG or other cannabinoids on AIM2 expression or activity, highlighting the originality of our results.

Cannabinoids have also been shown to modulate immune responses via the JAK/STAT pathway in T-lymphocytes through activation of the CB2 receptor (Xiong et al., 2022; Jiang et al., 2022b). Abnormal activation of the JAK/STAT pathway in RA has been associated with pathological processes of synovial inflammation and bone destruction (Qin et al., 2022; Simon et al., 2021; Hu et al., 2022). The Janus family of kinases (JAKs) comprises four non-receptor protein tyrosine kinases: JAK-1, JAK-2, JAK-3, and TyK-2 (tyrosine Kinase 2). These kinases act as dimers and signal transduction peptides upon phosphorylation, triggered by the binding of specific cytokines to their membrane-bound receptors (Babon et al., 2014a; Kameda, 2023). Once phosphorylated, JAK dimers recruit proteins from the signal transducer and activator of transcription (STAT) family, including STAT1–4, STAT-5A, STAT-5B, and STAT-6. After dimerization, STAT proteins translocate to the nucleus, where they regulate transcription of target gene, often by recruiting coactivators (Ivashkiv and Hu, 2004). This STAT-mediated transcription enhances cytokine production and other immune components, creating a positive feedback loop that perpetuates inflammation (Ivashkiv and Hu, 2004). Key cytokines regulated by the JAK/STAT pathway include INF-γ (via JAK-1, JAK-2, STAT-1, 3, 5), IL-6 (via gp130 receptor–JAK-1, JAK-2, TYK-2, STAT-1, 3, 5), IL-12 and IL-23 (via JAK-2, TYK-2 and STAT-3,4), GM-CSF (via JAK-2, STAT-5), and Receptor Activator of Nuclear Factor Kappa-Β Ligand (RANKL) (via IL6-gp 130 receptor–JAK-2/STAT-3 → IL-6, RANKL) (Hu et al., 2022; Kameda, 2023).

Although modulation of the JAK/STAT pathway has predominantly been studied in the context of cancer, one study has demonstrated that CBG modulates this pathway in a mouse model of atopic dermatitis (Jeong et al., 2025). Topical administration of CBG (0.1 mg/kg or 1 mg/kg) significantly reduced the protein levels of JAK-1, JAK-2, TYK-2, STAT-1, STAT-2, STA-3, p-STAT-3, STAT-6, p-STAT-6 (p < 0.05) and markedly decreased mRNA expression of pro-inflammatory cytokines including Tslp, IL-1β, IL-4, IL-6, IL-13, IL-17, IL-18, IL-22, and IL-33 (p < 0.001). Our results support the immunomodulatory and anti-inflammatory potential of CBG, although the extent to which these markers were modulated was less pronounced in our CIA model. This discrepancy could be attributed to differences in the molecular mechanisms of inflammation in different animal models, as well as variations in CBG dosage and posology. In our study, CBG after oral administration had a more pronounced effect on the systemic circulation (Figure 8), although mRNA levels of STAT-3 and STAT-5a were also affected in the synovial tissue. We have seen no marked changes in total STAT3-or p-STAT-3 protein levels following CBG treatment in the synovium in our WB analysis (Figure 9), which may reflect the protein stability and their longer half-life (reduced mRNA levels may not immediately translate to decreased protein abundance). Additionally, post-transcriptional regulatory mechanisms such as mRNA stability, translation efficiency, and microRNA-mediated repression can affect protein synthesis independently of mRNA expression, contributing to the lack of corresponding changes in STAT-3 protein levels (Vogel and Marcotte, 2012).

Our findings also suggest that GC exerts a broader suppressive effect on JAK-STAT signaling, both locally in synovial tissue and systemically (Figures 7, 8). This is consistent with established mechanisms of GC action. GC influence the JAK/STAT pathway in part through GR-mediated interference with downstream components of TLR signaling pathways, including inhibition of NF-κB, AP-1, and induction of MKP-1, as well as direct physical interaction of GR with STAT-3, STAT-5a, STAT-5b, and STAT-6 (Bhattacharyya et al., 2011).

GC also modulates JAK/STAT pathway by regulation of suppressors of cytokine synthesis (SOSCs), which are known to negatively affect this pathway (Chinenov and Rogatsky, 2007). It has been reported that GCs induced SOCS-1 expression with subsequent interfering with TLR-3 and TLR-4-mediated STAT-1 activation (Bhattacharyya et al., 2011). SOCS3, on the other hand, plays a critical role in regulating immune responses via its interaction with gp130, which is essential for the effective inhibition of IL-6/gp130-dependent STAT-3 activation–a major reason why we included it in our analysis (Babon et al., 2014b). In our study, GC treatment significantly reduced SOCS-3 mRNA expression, whereas CBG did not significantly alter SOCS-3 expression levels compared to the PCB group. This suggests that the modulation of STAT-3 is not mediated via the SOCS-3 regulatory pathway. Results of studies investigating the effects of cannabinoids on SOCS-3 expression vary across different cell types and conditions (for example, the selective CB2 receptor agonist, HU-308, facilitated SOCS-3 protein expression in a model of systemic sclerosis, whereas CBD reduced SOCS-3 protein expression in an intestinal epithelium model) (Tian et al., 2024; Koay et al., 2014).

IL-6 is a key pro-inflammatory cytokine closely associated with the activation of the JAK/STAT signaling pathway, particularly through STAT-3 and STAT-1 (Hu et al., 2021). Importantly, IL-6 also serves as a crucial factor that connects NF-κB and STAT-3 signaling pathways. While IL-6 and its receptor complex can effectively activate STAT-3, IL-6 itself is a known transcriptional target of NF-κB. Furthermore, STAT-3 plays a vital role in the activation of the NF-κB pathway, forming a positive feedback loop that amplifies inflammation (Hu et al., 2021). In our study both treatments significantly reduced STAT-3 mRNA expression, a statistically significant reduction of IL-6 mRNA expression was detected only in the GC-treated group in synovial tissue (Figure 4). Neither CBG nor GC showed a significant effect on IL-6 mRNA expression in blood samples (Figure 4), and no changes in IL-6 protein levels were observed by WB (Figure 9). We assume that this has been caused by high severity of the inflammation induced in our CIA model–evident from high clinical score and fact that also GC treatment induced only partial efficacy. Few studies support the conclusion that the effect of CBG leading to a measurable decrease of IL-6 gene or protein expression is achieved only in inflammation of mild to moderate intensity (Jeong et al., 2025).

In our *in vivo* model of CIA, CBG reduced TNF expression in the blood, achieving significance in half of the experiment, however no effect was seen in the synovial tissue. This fact is confirmed by the results of immunoblotting. Although previous research suggested that COX-2 could be another target for CBG another study in murine models of colitis as well as CIA did not find any influence of CBG on COX-2 expression (Sztolsztener et al., 2024; Borrelli et al., 2013). Our study supports the later findings.

Our findings suggest that CBG may also specifically target pyroptosis-related pathways, as evidenced by the significant reduction of gasdermin D mRNA levels in synovial tissue. These findings support limited evidence available for CBD, e.g., in a recent study that CBD induced downregulation of gasdermin D expression in a mouse model of oral ulcers (Qi et al., 2022).

In RA, synovial fibroblasts and immune cells (B-cells) exhibit elevated BCL-2 expression, contributing to apoptosis resistance and the persistent inflammation (Lee et al., 2013; Yang et al., 2016). Interestingly, our results indicate that neither CBG nor GC treatment reduces this anti-apoptotic marker; in fact, they led to increased BCL-2 mRNA expression. Although GC are known to induce apoptosis in cancer cells, RA-derived T cells are resistant to GC-induced apoptosis, associated with upregulation of BCL-2 (Makrygiannakis et al., 2008). Consistently, we also observed reduced expression of the pro-apoptotic BAX in the GC group, whereas no significant change in BAX mRNA expression was found in the CBG group compared to the PCB. Similarly, in an *in vitro* model of neuroinflammation, CBG was shown to inhibit apoptosis by preventing the increase in BAX level and reducing cleaved caspase-3 and increasing of BCL-2 expression (Gugliandolo et al., 2018). In the context of RA, this anti-apoptotic effect may be potentially detrimental, as resistance to apoptosis contributes to synovial hyperplasia and sustained inflammation (Baier et al., 2003). These findings suggest that while CBG may promote cell survival under inflammatory stress, its pro-survival properties could inadvertently exacerbate pathogenic synovial cell accumulation and chronic inflammation, highlighting a need for caution in therapeutic applications for RA.

The MAPK/ERK pathway is a signaling cascade that is essential for cell growth, differentiation, and survival (Thalhamer et al., 2008). It initiates a kinase cascade that includes Raf (MAPKKK), MEK1/2 (MAPKK), and ERK1/2 (MAPK) (Zhang and Liu, 2002). This pathway plays a central role in CIA model as well as RA in driving synovial inflammation and joint destruction (Thiel et al., 2007). Both CBG and GCs modulate the MEK/ERK signaling pathway, a driver of inflammation in RA, but they do so via distinct mechanisms. As a partial CB_2_ receptor agonist, CBG is believed to indirectly suppress ERK activation via CB_2_-mediated anti-inflammatory pathways, although direct data on ERK activation after CBG treatment in the CIA model are currently lacking. We acknowledge that this aspect could be more clearly addressed by including a CB_2_ receptor antagonist or conducting complementary *in vitro* experiments to confirm receptor involvement. (Navarro et al., 2018). We observed that the level of p-ERK/ERK was slightly influenced in the CBG group, though to a lesser extent than in the GC group. GCs exert a dual mechanism to inhibit ERK1/2 activation: they induce the expression of MAP kinase phosphatase-1 (MKP-1), which dephosphorylates ERK1/2, and simultaneously prevent MKP-1 degradation in immune cells like mast cells. However, in certain contexts, GCs fail to inhibit ERK1/2 due to continued proteasomal degradation of MKP-1 despite its upregulation (Kassel et al., 2001). Thus, although both CBG and GC may attenuate ERK signaling, CBG may provide a more durable, albeit less potent anti-inflammatory effect through stable CB2 engagement. The efficacy of GCs can fluctuate depending on cell-specific regulation of MKP-1 stability. This makes CBG a possible candidate for conditions where long-term modulation of ERK signaling could be beneficial.

RA associated joint destruction is accompanied by overexpression of several including matrix metalloproteinases (MMPs), particularly MMP-3 and MMP-9, which are considered as valid biomarkers of disease progression and joint destruction cytokines IL-23, IL-17, and chemokine RANKL (Bian et al., 2023; Ju et al., 2008; Adamopoulos et al., 2011). In our study, MMP-3 levels measured by qPCR, WB, and ELISA were consistent across methods. In the GC treatment group, serum levels of MMP-3, were significantly lower compared to the PCB group. Similarly, the relative mRNA expression of MMP-3, MMP-9, IL-17, IL-23 and RANKL in the synovial membrane was significantly reduced in the GC group. Although protein levels of MMP-3 showed only a decreasing trend, the overall data suggest that GC treatment effectively suppresses MMP expression, potentially limiting bone erosion and synovial inflammation. In contrast, there were no significant changes in MMP-3 levels in the CBG group, with only a trend towards decreased IL-17 expression in synovium and blood compared to the PCB group. Although a reduction in arthritis score might be expected to coincide with decreased MMP levels, our findings suggest that CBG’s beneficial effects may instead occur via mechanisms independent of MMP modulation. Also, the CBG effect was insufficient to diminish the enhancement of erosion and joint destruction related to the disease with highly induced inflammation and severity.

A limitation of the present study is that we investigated only the downstream inflammatory signaling pathways characteristic of the CIA model, without addressing the specific receptor-mediated mechanisms of CBG action. Moreover, all experiments were conducted *in vivo* using a rat model, and no complementary *in vitro* cellular assays were performed. Although the CIA model represents a well-established and compact system for studying rheumatoid arthritis–related inflammation, additional studies, including receptor-specific analyses and mechanistic *in vitro* and *in vivo* experiments, are required to fully elucidate the molecular targets and translational relevance of CBG.

## 5 Conclusion

Our study provides novel insights into the impact of CBG on inflammatory pathways in the CIA rat model. The effect of CBG was assessed by clinical scoring, paw width measurements, ELISA, and analysis of gene and protein expression of selected inflammatory markers in blood (collected at the midpoint and at the end of the experiment) and synovial membrane. CBG demonstrated a selective anti-inflammatory and immunomodulatory profile, notably through the downregulation of key signaling molecules such as TLRs, systemic NF-κB p65, STAT-3, and inflammasome-related components including NLRP1A, NLRP3, AIM2, gasdermin D, and caspase-1. It also reduced IL-1β and TNF expression during the early phase of disease and increased expression of the anti-apoptotic gene BCL-2.

Taken together, our findings indicate that CBG modulates distinct components of the inflammatory signaling pathways, and its effects translated into significant improvement in clinical scoring based on swelling, erythema and stiffness in rat CIA model.

## Data Availability

The raw data supporting the conclusions of this article will be made available by the authors, without undue reservation.
