# Supplementary material for: Regulation of inflammatory pathways by cannabigerol in the collagen induced arthritis model in rats
Source: Front Pharmacol. 2025 Oct 28;16:1705962. doi: 10.3389/fphar.2025.1705962 (PMC12602409; doi:10.3389/fphar.2025.1705962)
Supplement: Supplementary file 1 [file Table1.docx]

**Supplementary table S1.** Primer sequences.

| **name** | **sequence** | **NCBI Reference Sequences** |
| --- | --- | --- |
| **RPL13A F** | GGATCCCTCCACCCTATGACA | NM_173340 |
| **RPL13A R** | CTGGTACTTCCACCCGACCTC |  |
| **B2M F** | CGAGACCGATGTATATGCTTGC | NM_012512.2 |
| **B2M R** | GTCCAGATGATTCAGAGCTCCA |  |
| **HPRT1 F** | CCCAGCGTCGTGATTAGTGATG | NM_012583.2 |
| **HPRT1 R** | TTCAGTCCTGTCCATAATCAGTC |  |
| **PPIB F** | GGGCTCCGTTGTCTTCCTTT | NM_022536.2 |
| **PPIB R** | GACTTTAGGTCCCTTCTTCTTATCGTT |  |
| **IL-1β F** | CTC AAT GGA CAG AAC ATA AGC | NM_031512.2 |
| **IL-1β R** | GGT GTG CCG TCT TTC ATC A |  |
| **IL-6 F** | TCCTACCCCAACTTCCAATGCTC | NM_012589.2 |
| **IL-6 R** | TTGGATGGTCTTGGTCCTTAGCC |  |
| **IL-10 F** | GACAATAACTGCACCCACTTCC | NM_012854.2 |
| **IL-10 R** | GCATCACTTCTACCAGGTAAAACTTG |  |
| **IL-17A F** | CCATCCATGTGCCTGATGCT | NM_001106897.1 |
| **IL-17A R** | GTTATTGGCCTCGGCGTTTG |  |
| **TNFα F** | ATGGGCTCCCTCTCATCAGT- | NM_012675.3 |
| **TNFα R** | GCTTGGTGGTTTGCTACGAC |  |
| **COX-2 F** | AAGACAGATCAGAAGCGAGGAC | NM_017232.4 |
| **COX-2 R** | AGGATACACCTCTCCACCGAT |  |
| **Caspase 3 F** | GTGGAACTGACGATGATATGGC | NM_012922.2 |
| **Caspase 3 R** | CGCAAAGTGACTGGATGAACC |  |
| **VCAM-1 F** | ACTGTGACCTGTCAGCGAAG | NM_012889.2 |
| **VCAM-1 R** | TTAGGGACCGTGCAGTTGAC |  |
| **GM-CSF F** | CTCTGGAGAACGAAAAGAACGAAG | NM_053852.1 |
| **GM-CSF R** | TGGCTGGCTATCATGGTCAA |  |
| **NF-KB p65 (rela) subunit F** | ACCTGGAGCAAGCCATTAGC | NM_199267.2 |
| **NF-KB p65 (rela) subunit R** | AGTTCCGGTTTACTCGGCAG |  |
| **FOXP3** | AGTGCTAGAGCCAAGTGCTTC | NM_001108250.1 |
| **FOXP3** | GGAACAGGCCACTGTTAGGG |  |
| **MMP-3 F** | ATCCCTCTATGGACCTCCCAC | NM_133523.3 |
| **MMP-3 R** | AACAAGACTTCTCCCCGCAG |  |
| **MMP-9 F** | CCA CCG AGC TAT CCA CTC AT | NM_031055 |
| **MMP-9 R** | GTC CGG TTT CAG CAT GTT TT |  |
| **TLR2 F** | GGAAGCCCAAGAAAGCTCCT | NM_198769.2 |
| **TLR2 R** | GCTGGACCATGAGGTTCTCC |  |
| **TLR3 F** | AAA ACT GCC CGA GTC ACA GT | NM_198791.2 |
| **TLR3 R** | TGC ATC ATA GTC TAC TCC TTG CT |  |
| **TLR4 F** | GAT TGC TCA GAC ATG GCA GTT TC | NM_019178.2 |
| **TLR4 R** | CAC TCG AGG TAG GTG TTT CTG CTA A |  |
| **TLR5 F** | GGG CAG CAG AAA GAC GGT AT | NM_001145828.1 |
| **TLR5 R** | CAG GCA CCA GCC ATC CTT AA |  |
| **TLR7 F** | GTT TTA CGT CTA CAC AGT AAC TCT CTT CA | NM_001097582.1 |
| **TLR7 R** | TTC CTG GAG GTT GCT CAT GTT TT |  |
| **TLR8 F** | GAC CAC ACT GCA CTG CTA CT | NM_001101009.1 |
| **TLR8 R** | CAT GTT TTC CCC TTT CCG GC |  |
| **TLR9 F** | CCG AAG ACC TAG CCA ACC T | NM_198131.1 |
| **TLR9 R** | TGA TCA CAG CGA CGG CAA TT |  |
| **caspase 11 F** | ATG TGG AGA AGG ACT TCA TTG C | AY029283.1 |
| **caspase 11 R** | AGA TGA CAA GAG CAG GCA TGT A |  |
| **caspase 1 F** | CTG GAG CTT CAG TCA GGT CC | NM_012762.3 |
| **caspase 1 R** | CTT GAG GGA ACC ACT CGG TC |  |
| **NLRP3 F** | GTC CAG TGT GTT TTC CCA GAC | NM_001191642.1 |
| **NLRP3 R** | TGA GAA GAG ACC TCG GCA G |  |
| **JAK1 F** | AGG CAA GAG TGC ATA GAG CG | NM_001434542.1 |
| **JAK1 R** | TGG GAT CTC GCC ATT GTA GC |  |
| **JAK2 F** | CTC CAC AGA AGA AGA GGC CC | NM_031514.1 |
| **JAK2 R** | TTC AGA ACA TCG GCC TTC CC |  |
| **JAK3 F** | GAACCTCACATCTTGCTGCG | NM_012855.2 |
| **JAK3 R** | GCTCAACTTAGTCAGCGCAC |  |
| **SOCS-3 F** | CTA CTG GAG TGC CGT AAC CG | NM_053565.1 |
| **SOCS-3 R** | ATG CGT AGG TTC TTG GTC CC |  |
| **TYK2 F** | ACT ATG CAT CTG ATG TCT GGT CCT T | NM_001257347.1 |
| **TYK2 R** | CAG TCA TCT GGC CCT GGG TTA |  |
| **IL-23 P19 F** | ATA AGC ACC TGC TGG ACT CG | NM_130410.2 |
| **IL-23 P19 R** | GGA ACG GAG AAG AGA ACG CT |  |
| **RANKL F** | CCT GTA CTT TCG AGC GCA GA | AF187319.1 R |
| **RANKL R** | GTC GAG TCC TGC AAA CCT GT |  |
| **STAT1 F** | GACAAAGACCATGCCTTCGG | NM_032612.3 |
| **STAT1 R** | GAAGTCTAGAAGGGTGGACTTCA |  |
| **STAT2 F** | CGGCACTTTACCCTCAGAAG | NM_001011905.1 |
| **STAT2 R** | TGCCTAGTACAGCTTCCCTC |  |
| **STAT3 F** | AGAGGCGGCAGCAGATAGC | NM_012747.2 |
| **STAT3 R** | TTGTTGGCGGGTCTGAAGTT |  |
| **STAT4 F** | CCAGCCGTGCGAAGTTTCAA | NM_001012226.1 |
| **STAT4 R** | AAGGAGACTGTGGCTCCGTG |  |
| **STAT5a F** | CCGTGTCAGTTGTATCCCTG | NM_017064.2 |
| **STAT5a R** | TCAGCAATGACAGGTTCAGC |  |
| **STAT6 F** | TGCTCTTCTCTACGAGCTTCA | NM_001044250.1 |
| **STAT6 R** | ATGAACGATGACCACCAAGG |  |
| **BCL-2 F** | GGCCTTCTTTGAGTTCGGTG | U34964.1 |
| **BCL-2 R** | CACAGAGCGATGTTGTCCAC |  |
| **BAX F** | GCTAGCAAACTGGTGCTCAA | NM_017059.2 |
| **BAX R** | GGAAAGGAGGCCATCCCA |  |
| **Gasdermin-D F** | CTATTCAGCCCTCCCGGAAC | NM_001400993.1 |
| **Gasdermin-D R** | GCTGTCTGGTATAGTGGGGC |  |
| **NLRP1a F** | CAACAACAACAATCGGGAGACA | NM_001145755.2 |
| **NLRP1a R** | GCTGGGACAGTGGTAGGAAC |  |
| **AIM2 F** | TATGTTTTCCCTGGCCTGAA | NM_001427493.1 |
| **AIM2 R** | GGATTCGAGGCAGAATCACT |  |
| **NLRC4 F** | GCCCAATCTGCTGAAGCAAC | NM_001309432.1 |
| **NLRC4 R** | ATCCATCACTGCTCACACCG |  |

**Supplementary table S2.** Primary antibodies

| Primary antibody | Catalog number, Manufacturer | Dilution factor | Stock concentration | Final concentration [µg/mL] |
| --- | --- | --- | --- | --- |
| anti-ERK1/2 monoclonal antibody | #MA515134, Invitrogen, USA | 1:1,000 | 84 µg/mL | 0.084 |
| anti-phospho-ERK1/ERK2 (Thr202, Tyr204) monoclonal antibody | #MA5-1517, Invitrogen, USA | 1:1,000 | 52.0 µg/mL | 0.052 |
| anti-IL-6 Monoclonal Antibody | #ARC0962, Invitrogen, USA | 1:1,000 | 1 mg/mL | 1.00 |
| anti-TNF alpha Monoclonal Antibody | #60291-1-IG, Invitrogen, USA | 1:2,000 | 1.48 mg/mL | 0.74 |
| anti-MMP3 monoclonal antibody | #66338-1-IG, Invitrogen, USA | 1:1,000 | 1 mg/mL | 1.00 |
| anti-STAT3 monoclonal antibody | #MA113042, Invitrogen, USA | 1:1,000 | 1 mg/mL | 1.00 |
| anti-phospho-STAT3 (Tyr705) Monoclonal Antibody | #MA5-15193, Invitrogen, USA | 1:1,000 | 65 µg/mL | 0.065 |
| anti-beta2-microglobulin monoclonal antibody | #59035, Cell Signaling Technology, USA | 1:1,000 | 130 µg/mL | 0,130 |
